# Triaqua­bis­[4-(meth­oxy­carbon­yl)benzoato-κ*O* ^1^]zinc dihydrate

**DOI:** 10.1107/S1600536811010269

**Published:** 2011-03-23

**Authors:** Mihaela-Diana Şerb, Yutian Wang, Florina Dumitru, Ulli Englert

**Affiliations:** a Faculty of Applied Chemistry and Materials Science, University Politehnica of Bucharest, Polizu 1, RO-011061 Bucharest, Romania; b Institut für Anorganische Chemie, RWTH Aachen, Landoltweg 1, 52074 Aachen, Germany

## Abstract

In the crystal structure of the title complex, [Zn(C_9_H_7_O_4_)_2_(H_2_O)_3_]·2H_2_O, the Zn atom and the apical aqua ligand are located on a crystallographic twofold axis, with the Zn^II^ ion in a distorted square-pyramidal coordination geometry composed of five O atoms, two from the monodentate methyl­terephthalato group and three from water mol­ecules. The resulting complex and the two hydrate water mol­ecules are inter­connected by O—H⋯O hydrogen bonds.

## Related literature

For related Zn(II) complexes with terephtalato anions as ligands, see: Hawxwell *et al.* (2006 ▶); Li *et al.* (1998 ▶); Clausen *et al.* (2005 ▶); Sun *et al.* (2006 ▶); Yin *et al.* (2008 ▶); Carton *et al.* (2009 ▶). For hydrogen-bond motifs, see: Etter *et al.* (1990 ▶); Etter (1991 ▶). For a description of the coordination of the metal atom, see: Holmes (1984 ▶).
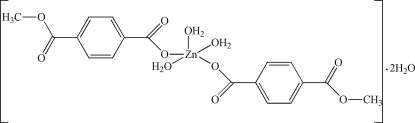


## Experimental

### 

#### Crystal data

[Zn(C_9_H_7_O_4_)_2_(H_2_O)_3_]·2H_2_O
*M* *_r_* = 513.74
Monoclinic, 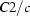

*a* = 13.7157 (15) Å
*b* = 5.9719 (7) Å
*c* = 25.874 (3) Å
β = 91.551 (2)°
*V* = 2118.5 (4) Å^3^
*Z* = 4
Mo *K*α radiation
μ = 1.23 mm^−1^
*T* = 130 K
0.28 × 0.17 × 0.02 mm

#### Data collection

Bruker SMART APEX CCD diffractometer
Absorption correction: multi-scan (*SADABS*; Bruker, 2004 ▶) *T*_min_ = 0.725, *T*_max_ = 0.976
12212 measured reflections
2435 independent reflections
2269 reflections with *I* > 2σ(*I*)
*R*_int_ = 0.045

#### Refinement

*R*[*F* ^2^ > 2σ(*F* ^2^)] = 0.032
*wR*(*F* ^2^) = 0.083
*S* = 1.06
2435 reflections
152 parameters
H-atom parameters constrained
Δρ_max_ = 0.43 e Å^−3^
Δρ_min_ = −0.30 e Å^−3^

### 

Data collection: *SMART* (Bruker, 2001 ▶); cell refinement: *SAINT-Plus* (Bruker, 1999 ▶); data reduction: *SAINT-Plus*; program(s) used to solve structure: *SHELXS97* (Sheldrick, 2008 ▶); program(s) used to refine structure: *SHELXL97* (Sheldrick, 2008 ▶); molecular graphics: *PLATON* (Spek, 2009 ▶); software used to prepare material for publication: *SHELXL97*.

## Supplementary Material

Crystal structure: contains datablocks global, I. DOI: 10.1107/S1600536811010269/nc2223sup1.cif

Structure factors: contains datablocks I. DOI: 10.1107/S1600536811010269/nc2223Isup2.hkl

Additional supplementary materials:  crystallographic information; 3D view; checkCIF report

## Figures and Tables

**Table 1:** Selected bond lengths (Å)

Zn1—O2	1.9763 (12)
Zn1—O2^i^	1.9765 (12)
Zn1—O5	2.003 (2)
Zn1—O6	2.0869 (14)
Zn1—O6^i^	2.0870 (14)

Symmetry code: (i) 
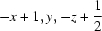
.

**Table 2:** Hydrogen-bond geometry (Å, °)

*D*—H⋯*A*	*D*—H	H⋯*A*	*D*⋯*A*	*D*—H⋯*A*
O5—H50⋯O1^ii^	0.88	1.78	2.6522 (16)	172
O6—H60⋯O10^iii^	0.82	1.96	2.763 (2)	170
O6—H61⋯O10	0.88	1.87	2.734 (2)	166
O10—H100⋯O1^iv^	0.82	1.94	2.741 (2)	166
O10—H101⋯O3^v^	0.83	1.92	2.7486 (19)	176

Symmetry codes: (ii) 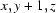; (iii) 
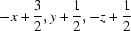
; (iv) 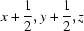; (v) 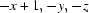.

**Fig. 1.**
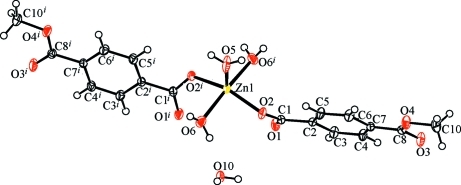
: PLATON (Spek, 2009) plot with displacement ellipsoids at 50% probability; H atoms are represented by spheres of arbitrary radius. Symmetry code: i = -x + 1, y, 1/2 - z

**Fig. 2.**
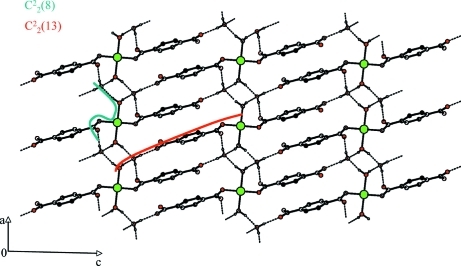
: Hydrogen-bond motifs in the title compound. The apical water molecule, the methyl substituents and H atoms attached to aryl groups have been omitted for clarity.

**Fig. 3.**
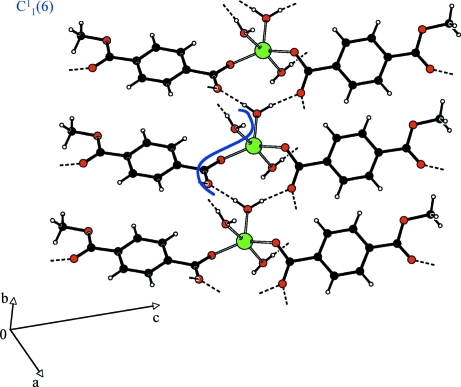
: Hydrogen-bond motifs in the title compound. The highlighted hydrogen bonds extend along b direction.

## supplementary crystallographic information

### Comment

The complex crystallizes in the space group *C*2/*c*, with half a molecule in the asymmetric unit. The angles are slightly distorted from regular square-pyramidal geometry and the Zn ion lies 0.3187 (3) Å above the basal plane. The fivefold coordination around the metal atom may be described as resulting from 65% of Berry pseudorotation from trigonal-bipyramidal to square pyramidal (Holmes, 1984). Selected bond distances are listed in Table 1. Packing in this solid is dominated by classical intermolecular O—H···O hydrogen bonding between the OH groups of the water molecules (donors of hydrogen bonds) and monoanions (acceptors of hydrogen bonds). All potential H donors find an acceptor in reasonable geometry for hydrogen bonding giving rise to C_2_^2^(8) motifs in the *a* direction, C_2_^2^(13) in the *ac* plane (Fig. 2) and C_1_^1^(6) in the *b* direction (Fig. 3) (Etter *et al.*, 1990; Etter, 1991). The hydrogen bond parameters are presented in Table 2. The shortest Zn···Zn separation amounts to 5.9719 (7) Å.

### Experimental

60 mg (2 mmol) Zn(NO_3_)_2_×6(H_2_O) and 40 mg (2 mmol) C_9_H_7_O_4_Na were stirred in 200 ml H_2_O at 50° C for 30 min. A white precipitate has formed, it has been removed by filtration. Slow evaporation of the solvent under ambient conditions gives crystals suitable for X-ray diffraction. Elemental analysis calcd (%): C 42.08, H 4.7, N 0; Found: C 41.54, H 4.94, N 0.

### Refinement

H atoms attached to oxygen were located from difference Fourier maps and treated as riding on the oxygen atoms with freely refined *U*_iso_. H atoms attached to carbon were calculated and introduced in their idealized positions with C_aryl_—H 0.95 Å, *U*_iso_(H) = 1.2*U*_eq_(C); C_methyl_—H 0.98 Å, *U*_iso_(H) = 1.5*U*_eq_(C).

### Crystal data

**Table:**

[Zn(C_9_H_7_O_4_)_2_(H_2_O)_3_]·2H_2_O	*F*(000) = 1064
*M**_r_* = 513.74	*D*_x_ = 1.611 Mg m^−^^3^
Monoclinic, *C*2/*c*	Mo *K*α radiation, λ = 0.71073 Å
Hall symbol: -C 2yc	Cell parameters from 2818 reflections
*a* = 13.7157 (15) Å	θ = 3.0–30.6°
*b* = 5.9719 (7) Å	µ = 1.23 mm^−^^1^
*c* = 25.874 (3) Å	*T* = 130 K
β = 91.551 (2)°	Plate, colorless
*V* = 2118.5 (4) Å^3^	0.28 × 0.17 × 0.02 mm
*Z* = 4	

### Data collection

**Table:**

Bruker SMART APEX CCD diffractometer	2435 independent reflections
Radiation source: fine-focus sealed tube	2269 reflections with *I* > 2σ(*I*)
graphite	*R*_int_ = 0.045
ω scans	θ_max_ = 27.5°, θ_min_ = 3.0°
Absorption correction: multi-scan (*SADABS*; Bruker, 2004)	*h* = −17→17
*T*_min_ = 0.725, *T*_max_ = 0.976	*k* = −7→7
12212 measured reflections	*l* = −33→33

### Refinement

**Table:**

Refinement on *F*^2^	Primary atom site location: structure-invariant direct methods
Least-squares matrix: full	Secondary atom site location: difference Fourier map
*R*[*F*^2^ > 2σ(*F*^2^)] = 0.032	Hydrogen site location: inferred from neighbouring sites
*wR*(*F*^2^) = 0.083	H-atom parameters constrained
*S* = 1.06	*w* = 1/[σ^2^(*F*_o_^2^) + (0.0415*P*)^2^ + 1.9928*P*] where *P* = (*F*_o_^2^ + 2*F*_c_^2^)/3
2435 reflections	(Δ/σ)_max_ < 0.001
152 parameters	Δρ_max_ = 0.43 e Å^−^^3^
0 restraints	Δρ_min_ = −0.30 e Å^−^^3^

### Special details

**Table:**

Geometry. All e.s.d.'s (except the e.s.d. in the dihedral angle between two l.s. planes) are estimated using the full covariance matrix. The cell e.s.d.'s are taken into account individually in the estimation of e.s.d.'s in distances, angles and torsion angles; correlations between e.s.d.'s in cell parameters are only used when they are defined by crystal symmetry. An approximate (isotropic) treatment of cell e.s.d.'s is used for estimating e.s.d.'s involving l.s. planes.
Refinement. Refinement of *F*^2^ against ALL reflections. The weighted *R*-factor *wR* and goodness of fit *S* are based on *F*^2^, conventional *R*-factors *R* are based on *F*, with *F* set to zero for negative *F*^2^. The threshold expression of *F*^2^ > σ(*F*^2^) is used only for calculating *R*-factors(gt) *etc*. and is not relevant to the choice of reflections for refinement. *R*-factors based on *F*^2^ are statistically about twice as large as those based on *F*, and *R*- factors based on ALL data will be even larger.

### Fractional atomic coordinates and isotropic or equivalent isotropic displacement parameters (Å^2^)

**Table:**

	*x*	*y*	*z*	*U*_iso_*/*U*_eq_	
Zn1	0.5000	0.22322 (5)	0.2500	0.01550 (11)	
O1	0.43857 (11)	−0.1990 (2)	0.16962 (5)	0.0234 (3)	
O2	0.50650 (10)	0.1406 (2)	0.17621 (5)	0.0206 (3)	
O3	0.30981 (11)	0.0349 (3)	−0.08830 (5)	0.0282 (3)	
O4	0.36264 (11)	0.3853 (2)	−0.07513 (5)	0.0256 (3)	
O5	0.5000	0.5587 (3)	0.2500	0.0363 (6)	
H50	0.4839	0.6475	0.2239	0.053 (9)*	
O6	0.65137 (10)	0.1991 (3)	0.25858 (6)	0.0263 (3)	
H60	0.6780	0.3069	0.2724	0.041 (8)*	
H61	0.6851	0.1665	0.2313	0.048 (8)*	
C1	0.45986 (13)	−0.0115 (3)	0.15193 (7)	0.0166 (4)	
C2	0.42900 (12)	0.0408 (3)	0.09683 (6)	0.0153 (4)	
C3	0.38816 (15)	−0.1255 (3)	0.06540 (7)	0.0207 (4)	
H3	0.3786	−0.2715	0.0789	0.025*	
C4	0.36134 (14)	−0.0791 (3)	0.01444 (7)	0.0207 (4)	
H4	0.3347	−0.1940	−0.0071	0.025*	
C5	0.44032 (15)	0.2554 (3)	0.07716 (8)	0.0209 (4)	
H5	0.4676	0.3701	0.0985	0.025*	
C6	0.41201 (15)	0.3030 (3)	0.02661 (8)	0.0218 (4)	
H6	0.4189	0.4506	0.0135	0.026*	
C7	0.37346 (13)	0.1352 (3)	−0.00510 (7)	0.0158 (4)	
C8	0.34468 (13)	0.1775 (3)	−0.06031 (7)	0.0178 (4)	
O10	0.77354 (10)	0.0523 (3)	0.18399 (5)	0.0229 (3)	
H100	0.8165	0.1421	0.1773	0.060 (10)*	
H101	0.7457	0.0254	0.1559	0.051 (8)*	
C10	0.33801 (16)	0.4409 (4)	−0.12870 (8)	0.0288 (5)	
H10A	0.3762	0.3468	−0.1517	0.043*	
H10B	0.3529	0.5989	−0.1350	0.043*	
H10C	0.2683	0.4142	−0.1355	0.043*	

### Atomic displacement parameters (Å^2^)

**Table:**

	*U*^11^	*U*^22^	*U*^33^	*U*^12^	*U*^13^	*U*^23^
Zn1	0.02039 (17)	0.01448 (17)	0.01142 (16)	0.000	−0.00319 (11)	0.000
O1	0.0324 (8)	0.0186 (7)	0.0188 (7)	−0.0025 (6)	−0.0073 (6)	0.0041 (5)
O2	0.0232 (7)	0.0263 (8)	0.0120 (6)	−0.0072 (6)	−0.0019 (5)	−0.0022 (5)
O3	0.0412 (8)	0.0253 (8)	0.0174 (7)	−0.0040 (7)	−0.0100 (6)	0.0009 (6)
O4	0.0369 (8)	0.0218 (8)	0.0176 (7)	−0.0032 (6)	−0.0065 (6)	0.0071 (6)
O5	0.0728 (17)	0.0133 (10)	0.0215 (10)	0.000	−0.0236 (10)	0.000
O6	0.0220 (7)	0.0342 (9)	0.0229 (7)	−0.0058 (6)	−0.0002 (6)	−0.0088 (6)
C1	0.0153 (8)	0.0200 (10)	0.0143 (8)	0.0017 (7)	−0.0015 (6)	−0.0008 (7)
C2	0.0147 (8)	0.0173 (9)	0.0137 (8)	0.0012 (7)	−0.0006 (6)	−0.0015 (7)
C3	0.0302 (10)	0.0141 (9)	0.0175 (9)	−0.0036 (8)	−0.0039 (7)	0.0021 (7)
C4	0.0266 (10)	0.0177 (10)	0.0174 (9)	−0.0041 (8)	−0.0055 (7)	−0.0015 (7)
C5	0.0297 (10)	0.0160 (9)	0.0169 (9)	−0.0050 (8)	−0.0041 (8)	−0.0024 (7)
C6	0.0326 (11)	0.0133 (9)	0.0194 (9)	−0.0033 (8)	−0.0029 (8)	0.0025 (7)
C7	0.0152 (8)	0.0181 (9)	0.0139 (8)	0.0004 (7)	−0.0008 (6)	0.0005 (7)
C8	0.0161 (9)	0.0215 (10)	0.0158 (9)	0.0026 (7)	0.0002 (7)	0.0010 (7)
O10	0.0238 (7)	0.0285 (8)	0.0160 (6)	−0.0015 (6)	−0.0046 (5)	0.0008 (6)
C10	0.0326 (11)	0.0325 (12)	0.0209 (10)	0.0001 (9)	−0.0053 (8)	0.0103 (9)

### Geometric parameters (Å, °)

**Table:**

Zn1—O2	1.9763 (12)	C2—C3	1.392 (3)
Zn1—O2^i^	1.9765 (12)	C3—C4	1.387 (2)
Zn1—O5	2.003 (2)	C3—H3	0.95
Zn1—O6	2.0869 (14)	C4—C7	1.388 (3)
Zn1—O6^i^	2.0870 (14)	C4—H4	0.95
O1—C1	1.247 (2)	C5—C6	1.384 (3)
O2—C1	1.268 (2)	C5—H5	0.95
O3—C8	1.208 (2)	C6—C7	1.390 (3)
O4—C8	1.324 (2)	C6—H6	0.95
O4—C10	1.456 (2)	C7—C8	1.493 (2)
O5—H50	0.88	O10—H100	0.82
O6—H60	0.82	O10—H101	0.83
O6—H61	0.88	C10—H10A	0.98
C1—C2	1.509 (2)	C10—H10B	0.98
C2—C5	1.389 (3)	C10—H10C	0.98
			
O2—Zn1—O2^i^	151.08 (9)	C2—C3—H3	119.8
O2—Zn1—O5	104.46 (4)	C3—C4—C7	119.94 (17)
O2^i^—Zn1—O5	104.46 (4)	C3—C4—H4	120.0
O2—Zn1—O6	90.85 (5)	C7—C4—H4	120.0
O2^i^—Zn1—O6	87.18 (6)	C6—C5—C2	120.29 (17)
O5—Zn1—O6	93.97 (4)	C6—C5—H5	119.9
O2—Zn1—O6^i^	87.17 (6)	C2—C5—H5	119.9
O2^i^—Zn1—O6^i^	90.85 (5)	C5—C6—C7	120.15 (18)
O5—Zn1—O6^i^	93.97 (4)	C5—C6—H6	119.9
O6—Zn1—O6^i^	172.07 (9)	C7—C6—H6	119.9
C1—O2—Zn1	128.49 (12)	C4—C7—C6	119.81 (17)
C8—O4—C10	116.67 (16)	C4—C7—C8	118.24 (17)
Zn1—O5—H50	127.0	C6—C7—C8	121.95 (17)
Zn1—O6—H60	115.1	O3—C8—O4	124.12 (17)
Zn1—O6—H61	118.4	O3—C8—C7	122.99 (18)
H60—O6—H61	106.7	O4—C8—C7	112.89 (16)
O1—C1—O2	125.53 (16)	H100—O10—H101	105.0
O1—C1—C2	118.06 (16)	O4—C10—H10A	109.5
O2—C1—C2	116.41 (16)	O4—C10—H10B	109.5
C5—C2—C3	119.45 (16)	H10A—C10—H10B	109.5
C5—C2—C1	120.40 (16)	O4—C10—H10C	109.5
C3—C2—C1	120.15 (17)	H10A—C10—H10C	109.5
C4—C3—C2	120.33 (18)	H10B—C10—H10C	109.5
C4—C3—H3	119.8		

Symmetry codes: (i) −*x*+1, *y*, −*z*+1/2.

### Hydrogen-bond geometry (Å, °)

**Table:**

*D*—H···*A*	*D*—H	H···*A*	*D*···*A*	*D*—H···*A*
O5—H50···O1^ii^	0.88	1.78	2.6522 (16)	172
O6—H60···O10^iii^	0.82	1.96	2.763 (2)	170
O6—H61···O10	0.88	1.87	2.734 (2)	166
O10—H100···O1^iv^	0.82	1.94	2.741 (2)	166
O10—H101···O3^v^	0.83	1.92	2.7486 (19)	176

Symmetry codes: (ii) *x*, *y*+1, *z*; (iii) −*x*+3/2, *y*+1/2, −*z*+1/2; (iv) *x*+1/2, *y*+1/2, *z*; (v) −*x*+1, −*y*, −*z*.
